# The *vitellogenin receptor* gene is involved in lifespan regulation of *Zeugodacus cucurbitae* (Coquillett) after short-term high-temperature treatment

**DOI:** 10.3389/fphys.2022.1090348

**Published:** 2022-12-22

**Authors:** Yuyang Lian, Sihua Peng, Xiaofeng Yang, Jingjing Jia, Jinlei Li, Aqiang Wang, Shuyan Yang, Rongjiao Zheng, Shihao Zhou

**Affiliations:** ^1^ Sanya Nanfan Research Institute of Hainan University, Sanya, China; ^2^ Key Laboratory of Germplasm Resources Biology of Tropical Special Ornamental Plants of Hainan Province, College of Forestry, Hainan University, Haikou, China; ^3^ Institute of Plant Protection, Hainan Academy of Agricultural Sciences (Research Center of Quality Safety and Standards for Agricultural Products of Hainan Academy of Agricultural Sciences), Haikou, China

**Keywords:** short-time high temperature, survival, lifespan, *Zeugodacus cucurbitae* (Coquillett), *vitellogenin receptor* gene

## Abstract

*Zeugodacus cucurbitae* (Coquillett) is a highly damaging agricultural pest in many tropical and subtropical countries around the world and high temperatures usually affect its survival. To clarify the effect of short-term high temperatures on the survival and lifespan of *Z. cucurbitae*, newly emerged adults of three consecutive generations (F_1_, F_2_, and F_3_) were exposed to 25 °C, 33 °C, 37 °C, 41 °C, or 45 °C treatments for 1 h. The effect of these temperatures on survival and lifespan was evaluated using biological indicators such as lifespan and pupation rate. Then, to study the molecular regulatory mechanism of the lifespan of *Z. cucurbitae* after short-term high-temperature treatment, we exposed the newly emerged adults to 25 °C or 45 °C treatments for 1 h and used siRNA to interfere with the expression of the *vitellogenin receptor* (*VgR*) gene in the female to study the effect of the *VgR* gene on the lifespan of *Z. cucurbitae*. The results showed that the survival rate, lifespan, pupae weight, pupation rate, and emergence rate of *Z. cucurbitae* decreased with increased temperature, while the female sex ratio of offspring increased. The heat resistance of females was higher than that of males. Interference with the expression of the *VgR* gene resulted in shortening of the female’s lifespan by approximately 60% after exposure to 25 °C or 45 °C treatments for 1 h, which indicated involvement of the *VgR* gene in the regulation of *Z. cucurbitae* lifespan. This study provides a reference to guide integrated control of *Z. cucurbitae* in high-temperature seasons.

## 1 Introduction

Temperature is an important environmental factor that has a remarkable influence on the survival, lifespan, and reproduction of insects because it can change the rate of chemical reactions and the spatial conformation of proteins (Amjad et al., 2022). The body temperature of insects is easily affected by the surrounding environment, and insects typically suffer from heat damage or even die when the environmental temperature exceeds the appropriate range for insect survival (Rinehart et al., 2000). The frequency and intensity of extremely high temperature events are increasing in the context of global warming (Ma and Ma, 2016). Insects typically encounter extremely high temperatures for short periods of time, rather than experiencing consistently high temperatures. In addition, extremely high temperatures may drive insect responses to climate change more than average temperatures (Ma et al., 2021). High mortality rates of 91.67% and 95% were observed for newly emerged adult *Grapholita molesta* Buscks exposed to 38 °C treatment for 48 h, and the lifespan decreased with increased temperature (Bao et al., 2019). Exposure of *Drosophila melanogaster* pupae to 40.5 °C treatment for 35 min resulted in curled, arched, and spherical shaped deformation in the wings of adults and a considerably reduced emergence rate (Williams et al., 2003). Exposure of *Bemisia tabaci* to 43–45 °C treatments for 1 h resulted in a remarkable increase in the proportion of female offspring (Cui et al., 2008). The differences in tolerance to high temperatures among different insects lead to species-specific effects of high-temperature stress on insect growth and development.

*Zeugodacus cucurbitae* (Coquillett) (Diptera: Tephritidae), commonly known as the melon fruit fly, is widely distributed in tropical and subtropical areas and is a pest for many crops (Puri et al., 2022). *Z. cucurbitae* is native to India and is now found in more than 40 countries around the world; it mainly harms the Cucurbitaceae family, which contains more than 125 host plants (Sajan et al., 2022). Adult females pierce the ovipositor into the fruit to lay eggs, and these eggs hatch into maggots that feed inside the fruit, thereby causing the fruit to decay. It is reported that *Z. cucurbitae* causes losses of up to 30–100% in different Cucurbitaceae crops. It has also been reported that female *Z. cucurbitae* lay eggs in unopened female and male flowers in Hawaii, and successful development of their larvae has been found in primary roots, stems, and petioles (Back and Pemberton, 1914). Therefore, determination of the effects of short-term high temperatures on the survival and lifespan of *Z. cucurbitae* could help clarify the timing of infestation during high-temperature seasons to guide the timely development of effective control strategies.

The embryonic development of *Z. cucurbitae* occurs by oviposition and mainly depends on the accumulation of sufficient yolk protein (YP) and other substances to support the development of oocytes and as essential nutrients for life (amino acids, proteins, lipids, phosphates, carbohydrates, ions, and vitamins) (Tufail and Takeda, 2009). Vitellogenin (Vg) is the most abundant YP precursor in insects, and it is transported to the membrane-bound vesicles of the oocyte *via* vitellogenin receptor (VgR)-mediated endocytosis, providing nutrients for embryonic development (Tufail and Takeda, 2005). Vg and VgR are inextricably linked with the reproduction of insects. Recently, it was demonstrated that Vg is a pleiotropic protein that not only provides nutrients for embryonic development, but also plays roles in stress resistance and antioxidation (Corona et al., 2007), climate adaptation (Amdam et al., 2005), activation of the ovary, regulation of lifespan, and wing differentiation (Page and Amdam, 2007). VgR, in concert with Vg, is involved in a range of physiological and behavioral activities in insects (Lian et al., 2022b). Our previous research showed that the reproductive ability and ovarian development rate of *Z. cucurbitae* were significantly decreased after interference with the *VgR* gene of 5-day-old females (Lian et al., 2022a). In addition, we found that interference with the *VgR* gene impacted the lifespan of female *Z. cucurbitae*; however, the mechanism of that effect was unclear. On this basis, we treated newly emerged adult *Z. cucurbitae* at 25 or 45 °C for 1 h, then injected siRNA into 5-day-old females to interfere with the expression of the *VgR* gene. Next, we reared them at room temperature and recorded the lifespans of females and males to assess the effects of interference with the *VgR* gene on the lifespan of *Z. cucurbitae* after short-term high-temperature treatment.

## 2 Materials and methods

### 2.1 Insect sources

Insects used in this experiment were obtained from balsam pear fields near Nada Town, Danzhou City, Hainan Province, China (109°29′ E, 19°30′ N), and reared in the laboratory on artificially prepared feed. Diet formulas for larvae and adults were prepared as described by Zeng et al. (2018), and both formulations were purchased from Hainan Qingfeng Biotechnology Co., Ltd (China). To generate a stable, temperature-sensitive population in the laboratory, the indoor rearing temperature for *Z. cucurbitae* was maintained at an average level of 25 ± 1 °C, the relative humidity was 70 ± 5%, and a 14 h/10 h light/dark cycle was used. The developmental stage of all insects exposed to short-term high temperatures in this study was newly emerged adult. All short-term treatments indicated in this study lasted one hour.

### 2.2 Setting of short-term high-temperature treatment

The *Z. cucurbitae* of the F_1_ generation (F_1_) used in this study were contemporary *Z. cucurbitae*, the *Z. cucurbitae* of the F_2_ generation (F_2_) were of the stage from eggs to adults derived from F_1_, and the *Z. cucurbitae* of the F_3_ generation (F_3_) were of the stage from eggs to adults derived from F_2_. It has been suggested that 25–30 °C is the optimal temperature range for the development and survival of *Z. cucurbitae* (Wei et al., 2011). Thus, a temperature greater than the highest temperature of the optimal range (>30 °C) in external environments was designated as high temperature (Zeng et al., 2019). Newly emerged adults of F_1_–F_3_ were exposed to 25 °C (control group), 33 °C, 37 °C, 41 °C, or 45 °C treatment for 1 h in an artificial climate chamber (Qianjiang Instruments, China). After treatment, all generational populations were reared at the standard indoor temperature (25 ± 1 °C). The survival rate (%) and lifespan (d) were recorded for each generation following completion of different temperature treatments. Each cage with 12 pairs (sex ratio, 12:12) was considered one replication, and six replicates were established for each treatment. The larvae of each generation were randomly selected and divided into 120 larvae per group, and six replicates were established for each treatment. The pupae weight (g), pupation rate (%), emergence rate (%), and sex ratio were recorded.

### 2.3 Design and preparation of siRNA

A 21-nucleotide siRNA was designed based on the conserved cDNA sequence of the *Z. cucurbitae VgR* gene, with a sense strand sequence of 5′-CGA​UGU​CGA​GGA​UGU​GUU​ATT-3′ and an antisense strand sequence of 5′-UAA​CAC​AUC​CUC​GAC​AUC​GTT-3′. The negative control (NC) was a commercially available siRNA, which was designed without homology to the target sequence and had no function as RNAi in any treatment, with a sense strand sequence of 5′-GGU​UCU​CCG​AAC​GUG​UCA​CGU-3′ and an antisense strand sequence of 5′-ACG​UGA​CAC​GUU​CGG​AGA​ACC-3'. Both siRNAs were synthesized by Qingke Biotechnology Co., Ltd. (China).

### 2.4 Injection of siRNA for *VgR* genes

Dry powder preparations of siRNA and the NC were resuspended at an initial concentration of 2.5 nmol and then diluted in tris-EDTA solution (125 μL) to prepare working solutions with a final concentration of 20 μM. The newly emerged *Z. cucurbitae* adults, after treatment at 25 °C or 45 °C for 1 h in an artificial climate box, were reared at the standard indoor temperature (25 °C ± 1 °C), as described above. On the 5th day after rearing, female adults were chosen for injection with 1.25 μg (4.5 μL) of siRNA or NC using a pneumatic microinjector (IM-11-2, NARISHIGE, Japan). The joint between the second and third segments of the abdominal backplane was the injection point. Injury treatment (using an empty injection needle) and control check (CK) groups were also prepared.

### 2.5 Effects of *VgR* gene interference on the lifespan of *Z. cucurbitae*

After injection, five female adults were isolated from each of the siRNA, NC, injury, and CK treatment groups, and each group was paired with five male adults, representing one replicate. Each treatment group was placed into a cage, with six replicates of each group prepared. Water, artificial adult feed, and pumpkin flakes were provided for rearing and reproduction, and cages were kept at an average temperature of 25 ± 1 °C. Lifespan data were then recorded.

### 2.6 Statistical analysis

The data were analyzed, in Excel (version 2022) and SPSS (version 26.0), using a script of completely randomized ANOVA and Tukey’s multiple comparisons. ANOVA was used after arcsine square root transformation of proportional data (Ahrens et al., 1990). The results presented in figures are the mean value ± standard error.

## 3 Results

### 3.1 Effects of short-term high temperature on the survival and lifespan of *Z. cucurbitae* (F_1_)

**FIGURE 1:**
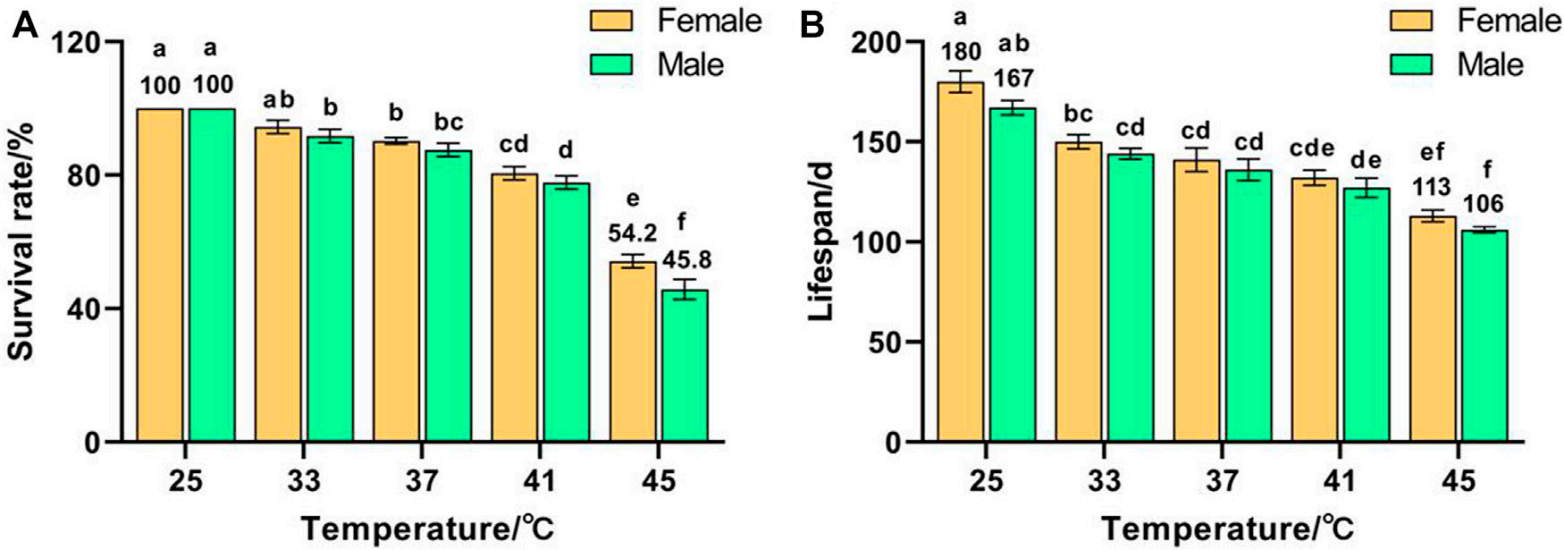
Effects of short-term high temperature on survival and lifespan of *Z. cucurbitae* (F_1_). **(A)** Survival rate. **(B)** Lifespan. The columns in the figure represent the mean ± standard error, and the letters above them indicate significant differences (*p* ≤ 0.05).

Temperature had a significant effect on the survival rate (*F* (9, 50) = 101.876, *p* < 0.001) and lifespan (*F* (9, 50) = 29.164, *p* < 0.001) of F_1_. The survival rates of females and males decreased with increased temperature and reached minima of 54.2% and 45.8%, respectively, for 45 °C treatment, which were significantly lower than those of the control groups (both were 100%) (Figure 1A). The lifespans of females and males decreased with increased temperature and reached minima of 113 and 106 days, respectively, for 45 °C treatment, which were significantly lower than those of the control groups (180 and 167 d, respectively) (Figure 1B). These results indicate that the survival rate and lifespan of *Z. cucurbitae* were adversely affected by short-term high temperature, and the survival rate and lifespan of females were higher than males exposed to the same treatment, suggesting that females were more heat tolerant than males.

### 3.2 Effects of short-term high temperature on the survival and lifespan of *Z. cucurbitae* (F_2_)

**FIGURE 2:**
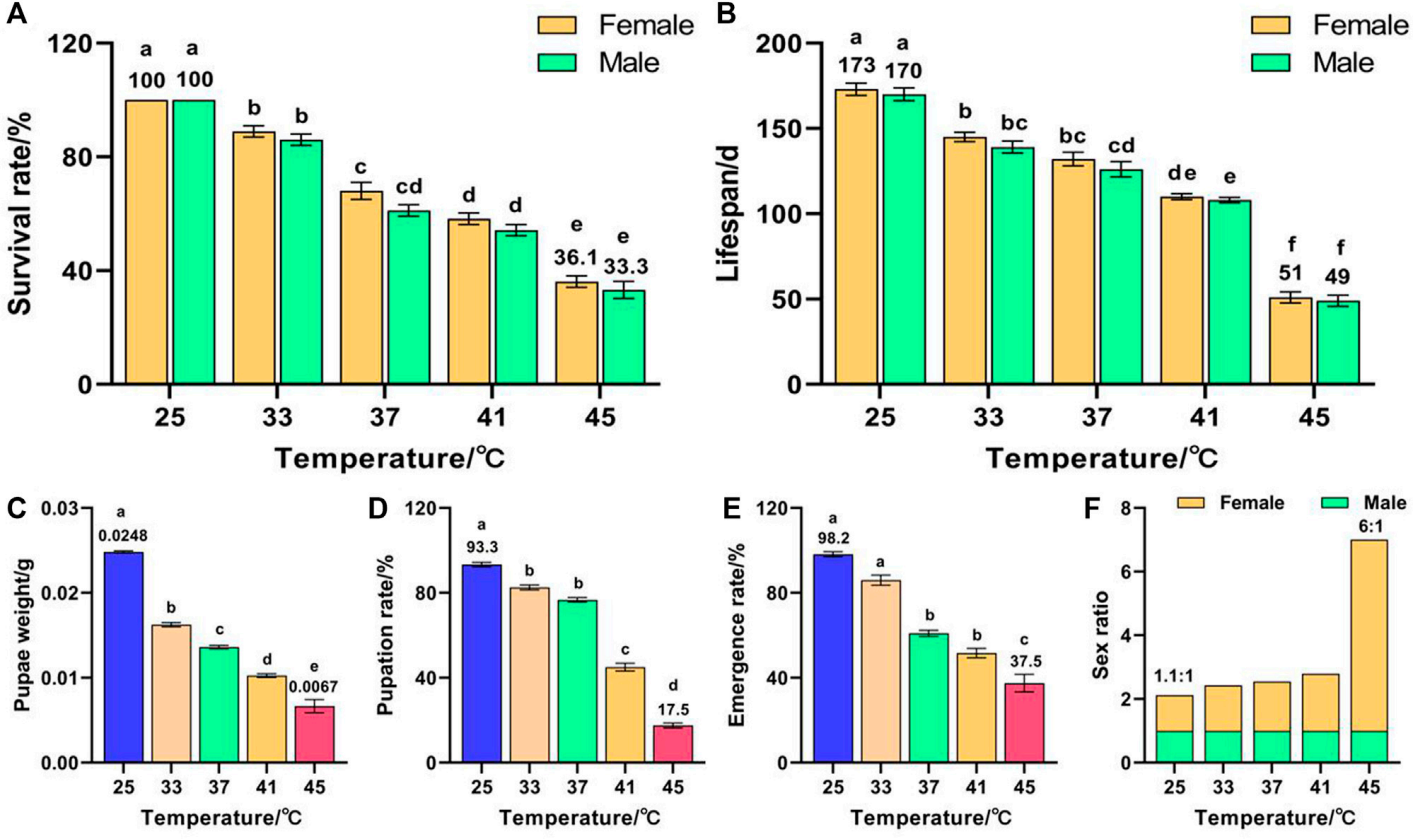
Effects of short-term high temperature on survival and lifespan of *Z. cucurbitae* (F_2_). **(A)** Survival rate. **(B)** Lifespan. **(C)** Pupae weight. **(D)** Pupation rate. **(E)** Emergence rate. **(F)** Sex ratio. The columns in the figure represent the mean ± standard error, and the letters above them indicate significant differences (*p* ≤ 0.05).

Temperature had a significant effect on the survival rate (*F* (9, 50) = 160.296, *p* < 0.001) and lifespan (*F* (9, 50) = 166.903, *p* < 0.001) of F_2_. The survival rates of females and males decreased with increased temperature and reached minima of 36.1% and 33.3%, respectively, for 45 °C treatment, which were significantly lower than those of the control groups (both were 100%) (Figure 2A). These results indicate that the females were more heat-resistant than the males in two consecutive generations of high-temperature treatment, and that the survival rate of F_2_ was lower than that of F_1_. The lifespans of females and males decreased with increased temperature and reached minima of 51 and 49 days, respectively, for 45 °C treatment, which were significantly lower than those of the control groups (173 and 170 days, respectively) (Figure 2B). In addition, temperature had a significant effect on the pupae weight (*F* (4, 373) = 490.347, *p* < 0.001), pupation rate (*F* (4, 25) = 541.36, *p* < 0.001), and emergence rate (*F* (4, 25) = 73.012, *p* < 0.001) of F_2_. The pupae weight (Figure 2C), pupation rate (Figure 2D), and emergence rate (Figure 2E) decreased with increased temperature and reached minima of 0.0067 g, 17.5%, and 37.5%, respectively, for 45 °C treatment, which were significantly lower than those of the control groups (0.0248 g, 93.3%, and 98.2%, respectively). The female sex ratio increased with increased temperature and reached a maximum of 6:1 for the 45 °C treatment (Figure 2F). These results indicate that, once again, females were more heat-resistant than males.

### 3.3 Effects of short-term high temperature on the survival and lifespan of *Z. cucurbitae* (F_3_)

**FIGURE 3:**
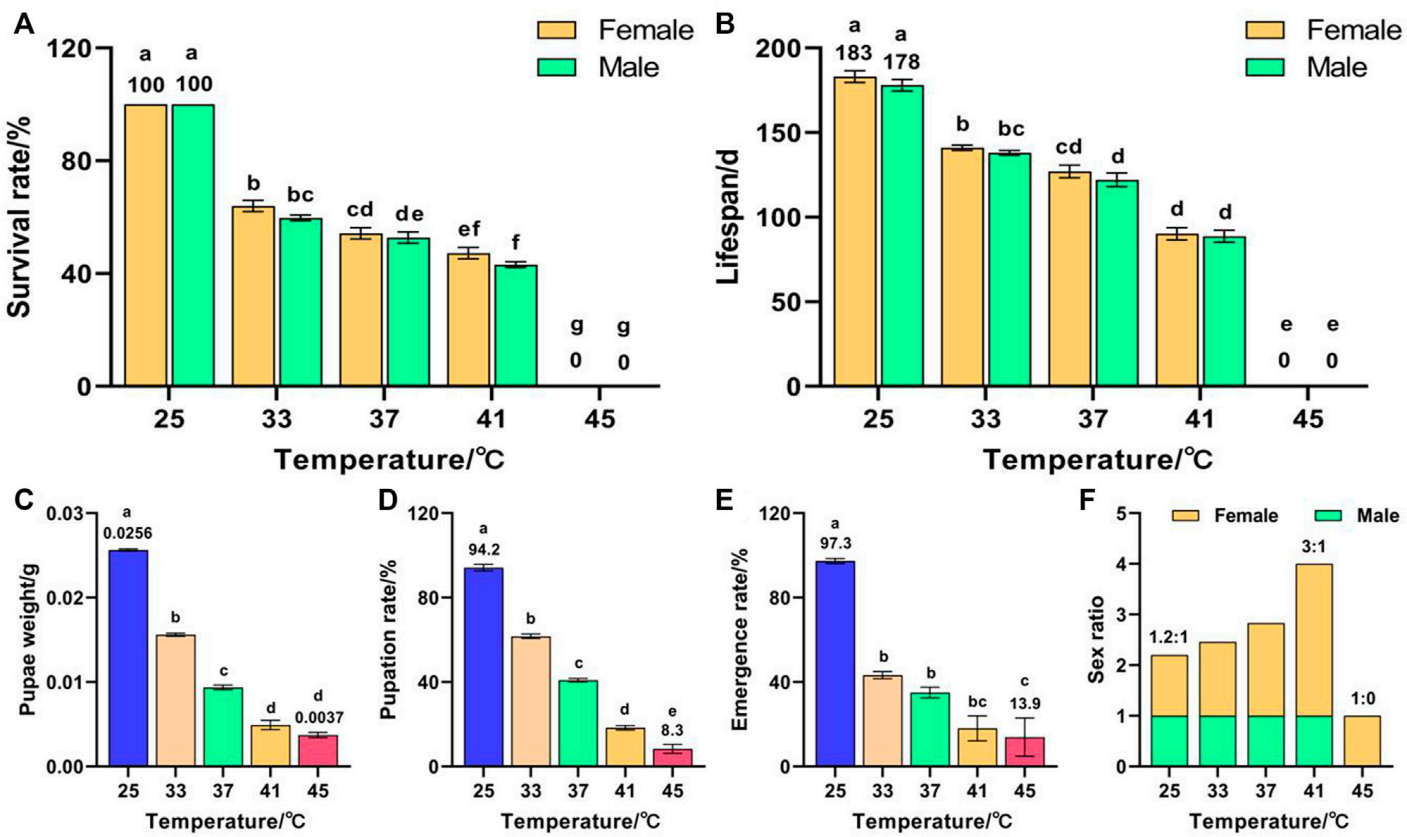
Effects of short-term high temperature on survival and lifespan of *Z. cucurbitae* (F_3_). **(A)** Survival rate. **(B)** Lifespan. **(C)** Pupae weight. **(D)** Pupation rate. **(E)** Emergence rate. **(F)** Sex ratio. The columns in the figure represent the mean ± standard error, and the letters above them indicate significant differences (*p* ≤ 0.05).

Temperature had a significant effect on the survival rate (*F* (9, 50) = 1623.853, *p* < 0.001), lifespan (*F* (9, 50) = 439.589, *p* < 0.001), pupae weight (*F* (4, 263) = 868.455, *p* < 0.001), pupation rate (*F* (4, 25) = 114.872, *p* < 0.001), and emergence rate (*F* (4, 25) = 14.22, *p* < 0.001) of F_3_. The survival rate decreased with increased temperature and all F_3_ died after exposure to 45 °C for 1 h (Figure 3A). The lifespans of females and males decreased with increased temperature and peaked in the control group at 183 and 178 days, respectively, which were significantly higher than those of the high-temperature treatment group (Figure 3B). The pupae weight (Figure 3C), pupation rate (Figure 3D), and emergence rate (Figure 3E) decreased with increased temperature and reached minima of 0.0037 g, 8.3%, and 13.9%, respectively, for 45 °C treatment, which were significantly lower than those of the control group (0.0256 g, 94.2%, and 97.3%, respectively). The female sex ratio increased with increased temperature (Figure 3F); lowest in the control group (1.2:1), highest in the 41 °C treatment group (3:1), and 1:0 in the 45 °C treatment group. These results indicate that exposure of both F_1_ and F_2_ to 45 °C treatment for 1 h led to a reduction in the number of F_3_ males, or even failure to complete emergence.

### 3.4 Effects of *VgR* gene interference on the lifespan of *Z. cucurbitae*

**FIGURE 4:**
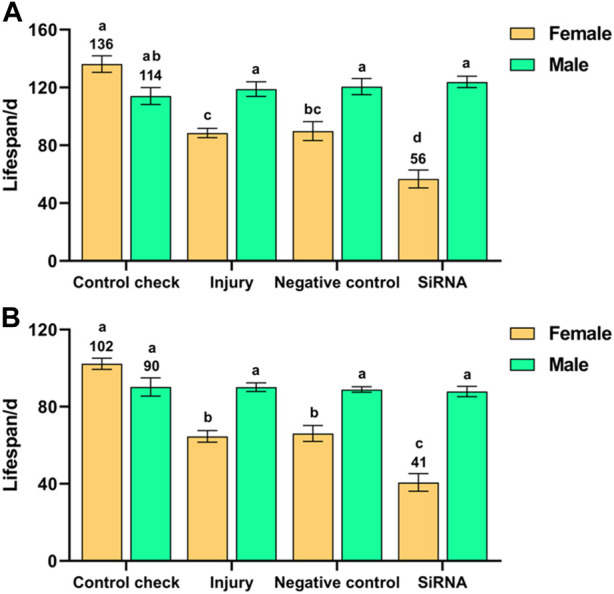
Effect of interference with the *vitellogenin receptor* gene on the lifespan of *Z. cucurbitae* after short-term high-temperature treatment. **(A)** Interference with the *vitellogenin receptor* gene after 25 °C treatment for 1 h. **(B)** Interference with the *vitellogenin receptor* gene after 45 °C treatment for 1 h. The columns in the figure represent the mean ± standard error, and the letters above them indicate significant differences (*p* ≤ 0.05).

Interference with the *VgR* gene had a significant effect on the lifespan of *Z. cucurbitae* after 5-day-old adults were exposed to 25 °C (*F* (7, 40) = 23.158, *p* < 0.001) or 45 °C (*F* (7, 40) = 34.952, *p* < 0.001) treatments for 1 h. There was no significant difference in the lifespan of males among the treatment groups after adults were exposed to 25 °C or 45 °C treatment for 1 h. The siRNA group presented the shortest lifespan among females (56 days) after adults were exposed to 25 °C treatment for 1 h, which was significantly lower than the other treatment groups; the lifespans of females in the injury and NC groups were significantly lower than that of the CK group (136 days). The lifespans of females were shorter than those of males in all treatment groups, except for the CK group, in which the lifespan of females was slightly higher than that of males (Figure 4A). The lifespan of females was highest in the CK group and lowest in the siRNA group (102 and 41 days, respectively) after adults were exposed to 45 °C treatment for 1 h, which were significantly different from other treatment groups (Figure 4B). In summary, interference with the *VgR* gene adversely affected the lifespan of females. The lifespan of the siRNA group was reduced by 59% and 60% at 25 °C and 45 °C, respectively, compared with the CK group.

## 4 Discussion

The effects of high temperatures on insects are mainly manifested *via* water loss, changes in intracellular ion concentration, disruption of the cytoskeleton, disruption of nerve conduction, and functional changes in biomolecules (proteins, DNA, etc.), thus causing insect mortality (Chown et a., 2011; Bowler, 2018). The survival rate and lifespan of *Z. cucurbitae* gradually decreased with increased temperature in our study. Gu et al. (2020) exposed *Z. cucurbitae* to high temperatures for 12 h and concluded that the survival rate of each insect state decreased continuously and the longevity of adults decreased continuously with increased temperature. This finding is consistent with the results of our study. He et al. (2019) subjected *Assara inouei* Yamanaka adults to short-term high temperatures and found that the survival rate and lifespan of adults decreased continuously with increased treatment temperature and treatment time, which is consistent with our results and indicates that the intensity and frequency of high temperature are closely related to the survival and lifespan of *Z. cucurbitae*. The survival rate and lifespan of *Z. cucurbitae* under the same treatment temperature decreased continuously with subsequent generations, and all of F_3_ died after exposure to 45 °C treatment for 1 h. These results demonstrate that the short-term high temperature not only affected the survival rate and lifespan of the contemporary *Z. cucurbitae*, but also reduced the heat tolerance of their offspring. Furthermore, the same high-temperature stress presented a higher lethality for the offspring than the contemporary. When exposed to the same high-temperature treatment time of 1 h, the survival rate and lifespan of *Z. cucurbitae* females were higher than males, indicating that females have better heat-resistance and adaptive capacity than males in response to short-term high temperature stress. This result is consistent with the analysis by Zhou (2016) of the effects of short-term temperature fluctuation on *Z. cucurbitae*, with females exhibiting a longer lifespan and higher survival rate than males.

The environmental conditions (photoperiod, temperature, and nutrition) experienced by the maternal insect affect offspring phenotype. This phenomenon is called the “maternal effect”, which is a non-genetic effect of the maternal environment on the offspring (Mousseau and Fox, 1998). The effects of short-term high temperatures on offspring were mainly reflected in decreased pupae weight, pupation rate, emergence rate, and increased female sex ratio in our study, which were consistent with the results of Zeng et al. (2022). The larvae of *B. tabaci* and *Heliothis virescens* also showed reduced pupae weights when they experienced sustained high-temperature stress at 35 °C (Ghazanfar et al., 2020). Studies have shown that heat injury can induce changes in the insect’s body size and that rapidly elevated temperatures usually result in enhanced metabolism and production of offspring, allowing for rapid population development (Cara et al., 2018; Tseng et al., 2018). Small offspring are more prone to dehydration and overheating than larger individuals (Gardner et al., 2011); however, they can reduce heat damage by promoting intestinal symbiotic bacteria to absorb water and nutrients (Kikuchi et al., 2016). Insects are able to regulate and mitigate the effects of high-temperature stress through phenotypic adaptations and rapid evolutionary responses (Bale et al., 2002). In our study, short-term high-temperature treatment reduced the pupae weight of offspring and produced smaller individuals as a way to improve resistance to high temperatures. The female sex ratio of the offspring increased with increased temperature, and the 45 °C treatment group of F_2_ possessed the largest female to male sex ratio of 6:1. In a study on the effect of different high-temperature treatments on the sex ratio of *Z. cucurbitae*, Gu et al. (2020) found that the female ratio of the offspring gradually increased with increased temperature after 12 h of high-temperature treatment, which is consistent with our results. Similar results have been reported in other insects. *Bradysia odoriphaga* adults were exposed to 37 °C treatment for 1 h, and the proportion of female offspring was remarkably higher than that of male offspring (Cheng et al., 2017). *Agasicles hygrophila* adults were exposed to 45 °C treatment for 1 h, and the female sex ratio of offspring was considerably increased (Zhao et al., 2009). In our study, newly emerged adults from F_2_ were exposed to 45 °C treatment for 1 h and their offspring (F_3_) were all females, with a sex ratio of 1:0, indicating that exposure to two consecutive high-temperature treatments causes a reduction in the number of F_3_ males, or even failure of males to complete emergence.

Vg and VgR are the basis of vitellogenesis, play crucial roles in the maturation of insect ovaries, and are potential targets for research on pest control (Lin et al., 2013). In recent years, an increasing number of studies have shown the pleiotropy of Vg and VgR. Vg and VgR are not only closely related to insect reproduction, but also involved in regulating a variety of physiological and behavioral activities (Wang et al., 2016). In our study, interference of the *VgR* gene resulted in a significant shortening of the lifespan of female *Z. cucurbitae* by approximately 60%, at 25 °C and 45 °C, indicating that silencing the *VgR* gene shortened the lifespan of female *Z. cucurbitae*. In insects, a circuit called IIS-JH-Vg/YP is present, in which the insulin/insulin-like growth factor 1 signaling (IIS) and TOR pathways mainly regulate lifespan and reproduction; they interact with the relevant juvenile hormone (JH) pathway to regulate reproductive activities such as vitellogenesis (Lin et al., 2021). Studies have shown that JH titers are low in IIS pathway-deficient *D. melanogaster* (Toivonen and Partridge, 2009). Studies of *D. melanogaster*, *Aedes aegypti*, *Tribolium castaneum*, and *Locusta migratoria* demonstrated that the synthesis of Vg requires the regulatory involvement of JH and that JH promotes the synthesis of Vg (Santos et al., 2019). Yamamoto et al. (2013) used removal of the pharyngeal lateral body of newly emerged adult *D. melanogaster* to reduce JH as a way to extend the lifespan of *D. melanogaster*, suggesting that the level of JH is negatively correlated with the lifespan of *D. melanogaster*. Therefore, for our study, we hypothesized that interference with the *VgR* gene would reduce VgR synthesis on the oocyte surface and that the ligand, Vg, would accumulate due to inability to enter the oocyte, thereby causing upregulation of JH and activation of the IIS pathway and reducing the lifespan of *Z. cucurbitae*. However, the molecular mechanism related to the effects of the *VgR* gene on lifespan in *Z. cucurbitae* requires further study.

In conclusion, we exposed newly emerged adult *Z. cucurbitae* to 25 °C, 33 °C, 37 °C, 41 °C, or 45 °C treatments for 1 h, and their biological indicators such as lifespan, pupation rate, and emergence rate were recorded. Our results showed that the survival rate, lifespan, pupae weight, pupation rate, and emergence rate of *Z. cucurbitae* decreased with increased temperature, the female sex ratio increased with increased temperature, and all of F_3_ died after exposure to 45 °C treatment for 1 h. Female *Z. cucurbitae* were more adaptable and resistant to short-term high-temperature stress than males. We also exposed newly emerged adult *Z. cucurbitae* to 25 °C or 45 °C treatment for 1 h, and then used siRNA to interfere with expression of the *VgR* gene in females. We found that down-regulation of the *VgR* gene caused a reduction in the lifespan of *Z. cucurbitae*. In future research, we aim to clarify the regulatory mechanisms involved in the effects of the *VgR* gene on the lifespan of *Z. cucurbitae*, establish the *Z. cucurbitae* transcriptome and proteome using high-throughput sequencing technology, and investigate the regulatory mechanism of the IIS-JH-Vg/YP circuit in *Z. cucurbitae*.

## Data Availability

The original contributions presented in the study are included in the article/Supplementary material; further inquiries can be directed to the corresponding author.
